# Phage VP882 possesses quorum-sensing-driven lytic induction and stress-mediated host growth suppression mechanisms

**DOI:** 10.64898/2026.07.27.740977

**Published:** 2026-07-27

**Authors:** Grace A. Beggs, Bonnie L. Bassler

## Abstract

Vibriophage VP882 launches its lytic cascade upon detection of a quorum-sensing autoinducer produced by its bacterial host. This capability enables the phage to maximize transmission by transitioning from lysogeny to lysis only at high host cell density, when abundant host cells are present to infect. Here, we show that two different pathways can be triggered upon prophage induction. When the prophage is induced via quorum sensing, Qtip, an anti-repressor, inhibits the cI lysogeny maintenance protein, driving expression of the lysis genes. The phage can also launch a cascade that causes host growth arrest. In this case, cI derepresses two genes, one encoding a DksA homolog, TraR _VP882_ , and one encoding a protein that we name QisA. TraR _VP882_ and QisA form a complex that causes host growth arrest, relying on RNAP-binding by TraR _VP882_ . Phage VP882 quorum sensing also activates production of a protein we call QtiQ, which inactivates the QisA-TraR _VP882_ complex, reestablishing host cell growth. Thus, in the presence of QtiQ, the phage lysis program is enacted. To our knowledge, QtiQ is the first protein inactivator of a TraR or DksA-like homolog. By inducing growth arrest, phage VP882 may enable its host to survive under stress-inducing conditions, or the phage may delay host lysis until optimal conditions are met. In both cases, the phage thereby enhances its own prospects for spread.

## Full Text Availability

The license terms selected by the author(s) for this preprint version do not permit archiving in PMC. The full text is available from the preprint server.

